# Reproducibility and Relevance of Acromial Morphology Measurements in Shoulder Pathologies: A Critical Review of the Literature

**DOI:** 10.3390/jcm14217760

**Published:** 2025-11-01

**Authors:** Marc Mombellet, Ramy Samargandi, Julien Berhouet

**Affiliations:** 1 Service de Chirurgie Orthopédique et Traumatologie, Centre Hospitalier Régional Universitaire (CHRU) de Tours, 37170 Tours, France; 2 Department of Orthopedic Surgery, College of Medicine, University of Jeddah, Jeddah 23218, Saudi Arabia

**Keywords:** acromial morphology, acromial tilt, acromial coverage, acromial height, posterior shoulder instability, shoulder osteoarthritis, rotator cuff tears

## Abstract

**Background:** The morphology of the acromion has long been implicated in shoulder pathology, particularly in relation to subacromial impingement and rotator cuff disease. More recently, interest has shifted toward the posterior acromion, with studies examining its potential role in posterior instability, eccentric glenohumeral osteoarthritis, and massive rotator cuff tears. **Methods:** A critical literature review of nine studies assessing sagittal acromial tilt, posterior coverage, and acromial height was conducted, emphasizing reproducibility and clinical significance across different shoulder disorders. **Results:** In posterior instability and eccentric osteoarthritis, the acromion is generally described as more horizontally oriented, less covering posteriorly, and positioned higher. Conversely, in massive cuff tears, it tends to appear more posteriorly covering without consistent change in tilt. Although these trends suggest a possible biomechanical role for the acromion, reported values vary widely between studies, and significant overlap exists between pathological and control groups. Such variability is compounded by differences in imaging modality, definitions of anatomical landmarks, and the frequent reduction of three-dimensional structures into two-dimensional projections. These methodological inconsistencies undermine reproducibility and limit the clinical applicability of posterior acromial parameters. **Conclusions:** Posterior acromial morphology appears to influence shoulder biomechanics, but existing measurements should be considered population-level markers rather than diagnostic thresholds. Future research should adopt standardized, three-dimensional, pathology-independent reference models anchored to stable scapular landmarks and validated across imaging modalities to improve reproducibility and clinical utility.

## 1. Introduction

The acromion represents the lateral and anterior extension of the scapular spine, articulating with the clavicle and forming, together with the coracoacromial ligament, the superior osteofibrous arch that partially covers the humeral head. Its anterior morphology, through the analysis of its sagittal inclination, has long been studied in the context of subacromial impingement and rotator cuff tears (RCTs). Early studies suggested that variations in sagittal acromial orientation could predispose degenerative tendon tears, with a more vertical acromial tilt being associated with increased risk of RCTs [1,2,3,4]. Nevertheless, the causal relationship between acromial morphology and rotator cuff disease has remained debated, and the reproducibility of such measurements has often been questioned.

More recently, attention has shifted from the anterosuperior aspect of the acromion to its posterior morphology. A growing body of research has explored the role of posterior acromial parameters—namely sagittal acromial tilt, posterior acromial coverage, and acromial height—in chronic shoulder pathologies beyond impingement. These parameters have been investigated in the context of posterior glenohumeral instability and eccentric (posteriorly subluxating) glenohumeral osteoarthritis, where a more horizontal, less covering, and higher acromion has been described. In contrast, in massive RCTs, distinct adaptations have been reported, including a more downwardly inclined and more posteriorly covering acromion.

Despite these intriguing associations, the literature remains heterogeneous and sometimes contradictory. Reported differences between pathological and control groups, although frequently significant, are often accompanied by wide standard deviations and overlapping ranges of values, limiting their discriminatory power at the individual level. Moreover, the methodologies used across studies are highly variable. Imaging modalities range from plain radiographs to MRI and 3D reconstructions from CT or MRI, and reference landmarks differ substantially [5,6,7,8,9,10,11,12]. While some authors rely on the glenoid center as a fixed point, this structure may itself be altered by pathology, such as posterior erosion in eccentric osteoarthritis or retroversion in posterior instability. Such variability raises important questions about the reproducibility and validity of these parameters.

Another challenge lies in the dimensionality of assessment. Many studies have reduced complex three-dimensional scapular anatomy to two-dimensional planes, potentially distorting angular or linear measurements. Even small differences in scapular orientation can alter the results significantly [13]. Consequently, planar methods may fail to reflect the true spatial relationship between the acromion, glenoid, and humeral head. The absence of standardized, pathology-independent reference systems further compromises comparability and clinical applicability.

Accordingly, the objective of this critical review is to synthesize current evidence on posterior acromial morphology and assess the reproducibility and clinical significance of key parameters: sagittal tilt, posterior coverage, and acromial height, across major shoulder pathologies. Specifically, we aimed to determine whether consistent morphologic trends exist and to what extent methodological heterogeneity limits their diagnostic or biomechanical interpretation.

## 2. Method

To critically appraise the role of posterior acromial morphology in shoulder pathology, we conducted a literature search across PubMed, Scopus, and Web of Science using the terms acromial tilt, acromial coverage, acromial height, posterior shoulder instability, shoulder osteoarthritis, rotator cuff tears. No restrictions were applied for publication date or language in order to maximize capture. The primary aim was not exhaustive retrieval but rather a focused critical synthesis of studies reporting quantitative acromial measurements in clinically relevant populations. Because this work was conceived as a critical narrative review rather than a systematic review, it did not follow PRISMA guidelines. Formal risk-of-bias assessment was not applicable, as the focus was on methodological appraisal rather than quantitative synthesis.

Inclusion criteria were studies reporting at least one quantitative parameter of posterior acromial morphology, specifically sagittal acromial tilt, posterior acromial coverage, or acromial height. Eligible articles had to provide a clear methodological description of how the measurement was defined and obtained, as well include comparisons between pathological shoulders (posterior shoulder instability, eccentric glenohumeral osteoarthritis, or massive rotator cuff tears), and appropriate control or comparator groups. Both radiographic modalities (plain radiographs, MRI) and tomographic methods (CT scans, 3D reconstructions) were considered. Exclusion criteria included the absence of explicit measurement methodology, purely qualitative or descriptive studies, and investigations of acromial morphology not directly related to instability, osteoarthritis, or cuff pathology.

Following screening of titles, abstracts, and full texts where necessary, nine studies were retained for critical review. These can be grouped into three major clinical contexts:

- Posterior shoulder instability: five studies analyzed sagittal acromial tilt, posterior coverage, and/or acromial height (Meyer et al., 2019 [5]; Arner et al., 2023 [6]; Livesey et al., 2023 [7]; Beeler et al., 2021 [8]; Akgun et al., 2024 [9]).
- Eccentric (posteriorly subluxation) glenohumeral osteoarthritis: three studies evaluated sagittal tilt and posterior coverage (Meyer et al., 2019 [10]; Beeler et al., 2018 [11]; Verhaegen et al., 2021 [12]).
- Massive RCTs: one study compared posterior coverage and tilt between cuff tears and concentric osteoarthritis (Beeler et al., 2018 [14]).

The data were then analyzed according to each morphological parameter, sagittal acromial tilt, posterior coverage, and acromial height, with both intra-study and inter-study comparisons of values, methodologies, and imaging modalities. This parameter-based structure allows identification of morphologic trends while simultaneously highlighting methodological variability that undermines reproducibility and clinical interpretation.

## 3. Results from the Literature

### 3.1. Posterior Shoulder Instability

#### 3.1.1. Sagittal Acromial Tilt

**Figure 1:**
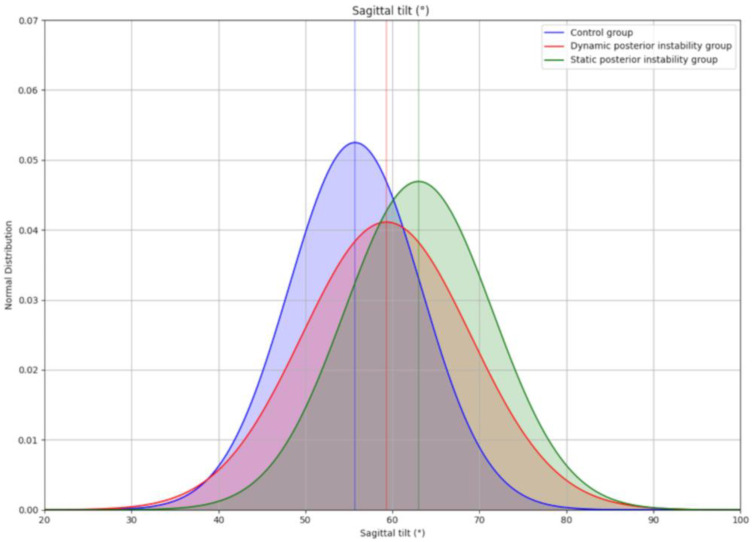
Schematic representation of the overlap between the different groups in the study by Beeler et al., 2021 [8], concerning the sagittal inclination parameter. Considering that the distributions follow a normal distribution with μ and σ being the mean and standard deviation, respectively. Control group: N (μ = 55.7, σ = 7.6), dynamic posterior instability group: N (μ = 59.3, σ = 9.7), static posterior instability group: N (μ = 63.0, σ = 8.5).

Most studies report a more horizontal acromion in posterior instability. Meyer et al., 2019 [5] found greater tilt in posterior instability than in controls and anterior instability (63.6° ± 9.8 vs. 55.9° and 55.8°; *p* < 0.001). Beeler et al., 2021 [8] confirmed this with CT-based reconstructions, reporting 63.0° ± 8.5 in static instability compared with 55.7° ± 7.6 in controls (*p* = 0.004). In our analysis, we illustrated the substantial overlap between pathological and control distributions (Figure 1), underlining the limited discriminatory power of tilt values. Akgun et al., 2024 [9] reported an even greater difference, with 78.8° ± 5.0 in constitutional static instability (C1) compared to 68.8° ± 3.9 in controls (*p* < 0.001). Livesey et al., 2023 [7] demonstrated progressive increases in tilt with greater posterior glenoid bone loss (58.5° → 64.3° → 67.7°). In contrast, Arner et al., 2023 [6] observed lower tilt values in instability (55.2° ± 9.6) compared with controls (62.2° ± 13.7; *p* = 0.03). These results are collated in Table 1 and compared across series in Table 2.

#### 3.1.2. Posterior Acromial Coverage

Coverage is generally reduced in posterior instability. Meyer et al., 2019 [5] reported 48.8° ± 8.5 in posterior instability compared with 61.6° in controls (*p* < 0.001). Beeler et al., 2021 [8] found reduced coverage in static instability (54.6° ± 6.7) and dynamic instability (56.7° ± 9.5) compared with controls (62.9° ± 7.5; *p* ≤ 0.007). Akgun et al., 2024 [9] reported 52.8° ± 11.1 in instability vs. 63.2° ± 6.3 in controls (*p* = 0.002). Livesey et al., 2023 [7] observed significant differences only when pooling patients with bone loss (~60.3° vs. 65.4°; *p* < 0.05). Arner et al., 2023 [6] found markedly lower coverage in instability (68.3° ± 11) compared with controls (81.7° ± 19.1; *p* < 0.05). The variability in absolute values is summarized in Table 2.

#### 3.1.3. Acromial Height

Most studies suggest increased acromial height in posterior instability. Meyer et al., 2019 [5] reported 30.9 ± 6.7 mm vs. 20.4 mm in controls (*p* < 0.001). Beeler et al., 2021 [8] observed 21.3 ± 4.2 mm in static instability vs. 15.5 ± 4.9 mm in controls (*p* < 0.001). Akgun et al., 2024 [9] found 21.9 ± 4.6 mm in instability vs. 18.6 ± 4.7 mm in controls (*p* = 0.001). Livesey et al., 2023 [7] reported a stepwise increase with bone loss (17.6 → 20.4 → 21.9 mm; *p* = 0.021). In contrast, Arner et al., 2023 [6] reported lower absolute values overall (11 mm in instability vs. 0.7 mm in controls), likely reflecting different measurement definitions. Comparative data are presented in Table 2.

### 3.2. Eccentric (Posteriorly Subluxating) Glenohumeral Osteoarthritis

#### 3.2.1. Sagittal Acromial Tilt

A more horizontal tilt is consistently reported in eccentric osteoarthritis. Meyer et al., 2019 [10] found tilt of 61° ± 9 in B2–C glenoids vs. 56° ± 11 in concentric OA (*p* = 0.004). Beeler et al., 2018 [11] reported 72° ± 7.9 in eccentric OA vs. 67° ± 7.3 in concentric OA (*p* = 0.002). Verhaegen et al., 2021 [12] confirmed this trend, with 70° ± 9 in OA shoulders vs. 64° ± 8 in controls (*p* < 0.001).

#### 3.2.2. Posterior Acromial Coverage

Coverage is generally reduced in eccentric OA. Beeler et al., 2018 [11] found 53° ± 6.9 in eccentric OA vs. 57° ± 5.8 in concentric OA (*p* = 0.01). Verhaegen et al., 2021 [12] reported 50° ± 8 in OA vs. 55° ± 7 in controls (*p* < 0.05). Meyer et al., 2019 [10] did not report coverage.

#### 3.2.3. Acromial Height

Not evaluated in these studies.

Findings are summarized in Table 2, showing consistent trends but wide ranges.

### 3.3. Massive Rotator Cuff Tears

#### 3.3.1. Sagittal Acromial Tilt

Beeler et al., 2018 [14] reported no significant difference in tilt between cuff tear patients and concentric OA (64.5° ± 7 vs. 67.2° ± 7.5).

#### 3.3.2. Posterior Acromial Coverage

The same study found greater posterior coverage in cuff tears (60.0° ± 8.6) compared with concentric OA (54.5° ± 5.9; *p* = 0.01) [14].

#### 3.3.3. Acromial Height

Not reported in this study.

Results for cuff tears are summarized in Table 2.

### 3.4. Comparative Overview

Across pathologies, posterior instability and eccentric OA tend to show a more horizontal, less covering, and higher acromion, while massive cuff tears are associated with greater posterior coverage. However, as shown in Table 1 and Table 2, substantial variability, wide standard deviations, and inconsistent definitions across studies prevent the establishment of reliable pathological thresholds. A synthesis of these overall trends is presented in Table 3, which provides a concise summary of the characteristic acromial patterns observed in each condition.

## 4. Critical Synthesis and Methodological Appraisal

**Figure 2:**
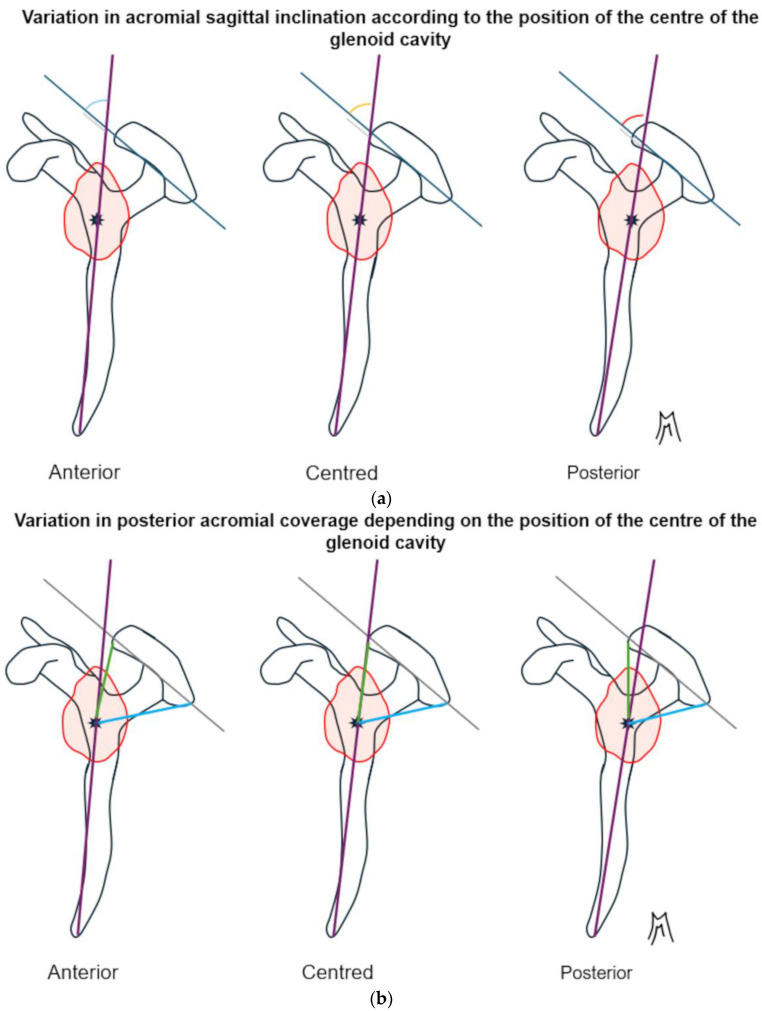

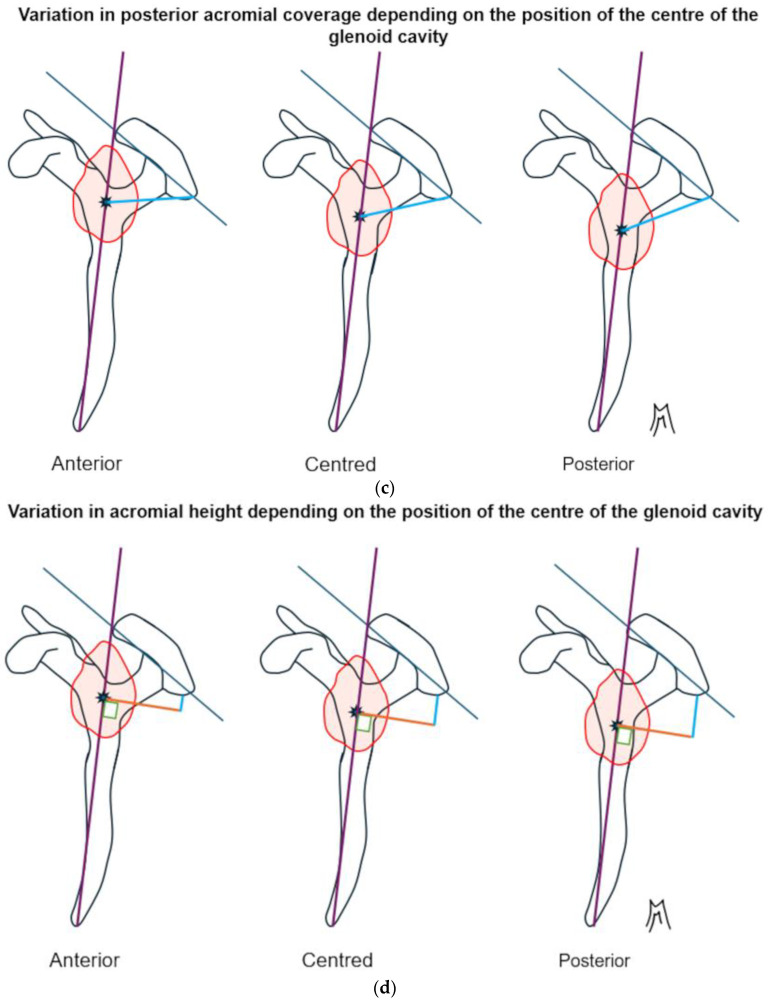
Schematic representation of a lateral view of the left scapula. The coracoid process is located on the left, and the acromion is on the right and above. The glenoid cavity is represented by the red circle. The center of the glenoid cavity is represented by the black asterisk. (**a**) The more posterior the position of the center of the glenoid cavity, which serves as a reference point for the vertical axis, the more open the sagittal tilt angle is. Consequently, a more posterior center of the glenoid cavity gives the impression that the acromion is more horizontal. (**b**) The more posterior the position of the center of the glenoid cavity, which serves as a reference point for the vertical axis, the more the posterior coverage angle decreases and the anterior coverage angle increases. Consequently, the anteroposterior position of the center of the glenoid cavity influences the coverage by the acromion. (**c**) The higher the position of the center of the glenoid cavity, which serves as a reference point for the vertical axis, the greater the posterior coverage angle and the greater the total coverage. Consequently, the height of the position of the center of the glenoid cavity influences the coverage by the acromion. (**d**) The higher the position of the center of the glenoid cavity, which serves as a reference point for the vertical axis, the lower the acromial height. Consequently, the height of the position of the center of the glenoid cavity influences the perception of the height of the acromion.

**Figure 3:**
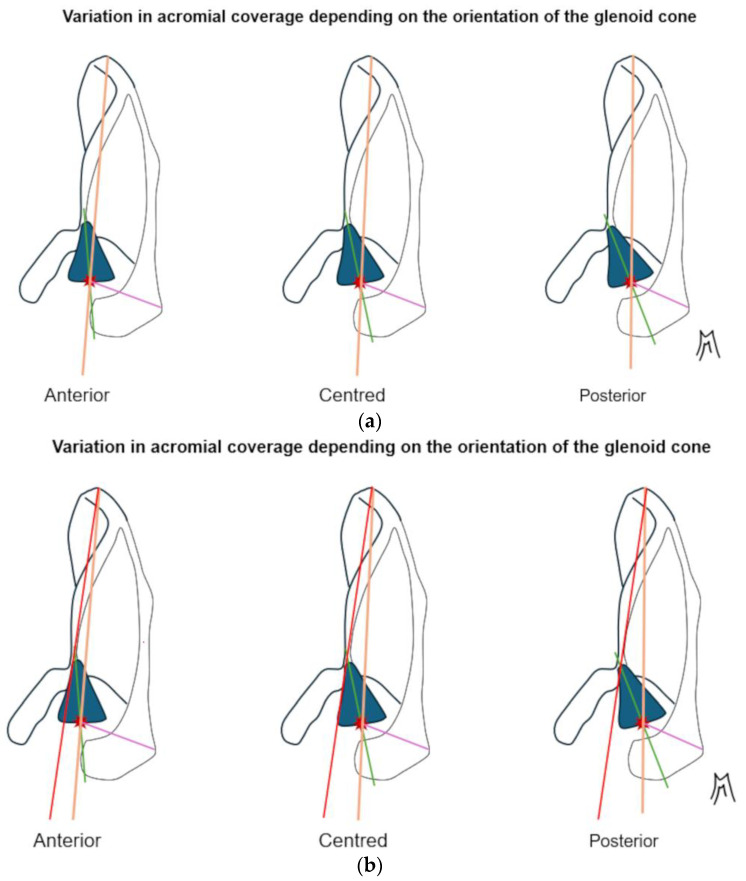
Schematic representation of a superior view of the left scapula. The coracoid process is located on the left and the scapular spine on the right. The glenoid cone is represented by the blue cone and the center of the glenoid cavity by the red asterisk. (**a**) The further the glenoid cone tilts backwards, the more posterior the center of the glenoid cavity becomes and the more the posterior acromial coverage decreases. The Friedman axis does not take this posterior tilt into account. (**b**) Here, we see an increase in the angle between the red axis, which is fixed and passes through the medial scapular angle and the anterior part of the scapular notch, and the green axis, which is fixed and bisects the glenoid cone. This supports the hypothesis of the existence of a pivot point at the base of the glenoid cone.

**Figure 4:**
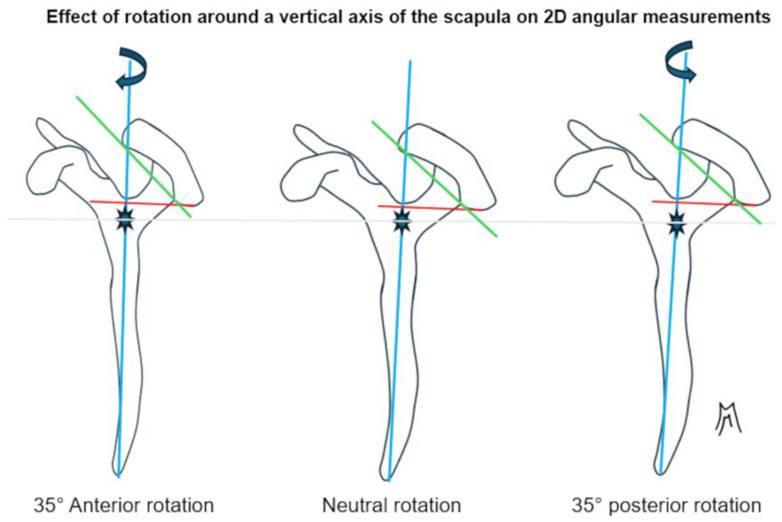
Schematic representation of a lateral view of the left scapula. The coracoid process is located on the left, and the acromion is on the right and above. The center of the glenoid cavity is represented by the blue asterisk. Posterior rotation along the vertical axis gives the impression that the acromion is higher and more horizontal. This is a 2D diagram, and it does not take into account the superimposition of reliefs, which limits interpretation.

**Figure 5:**
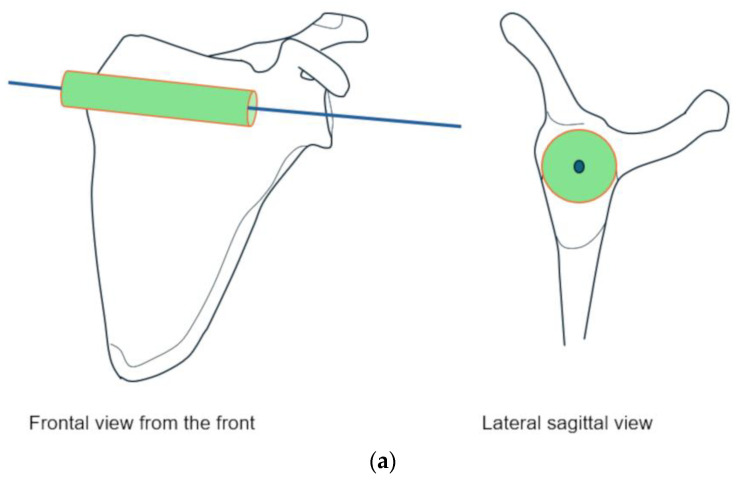

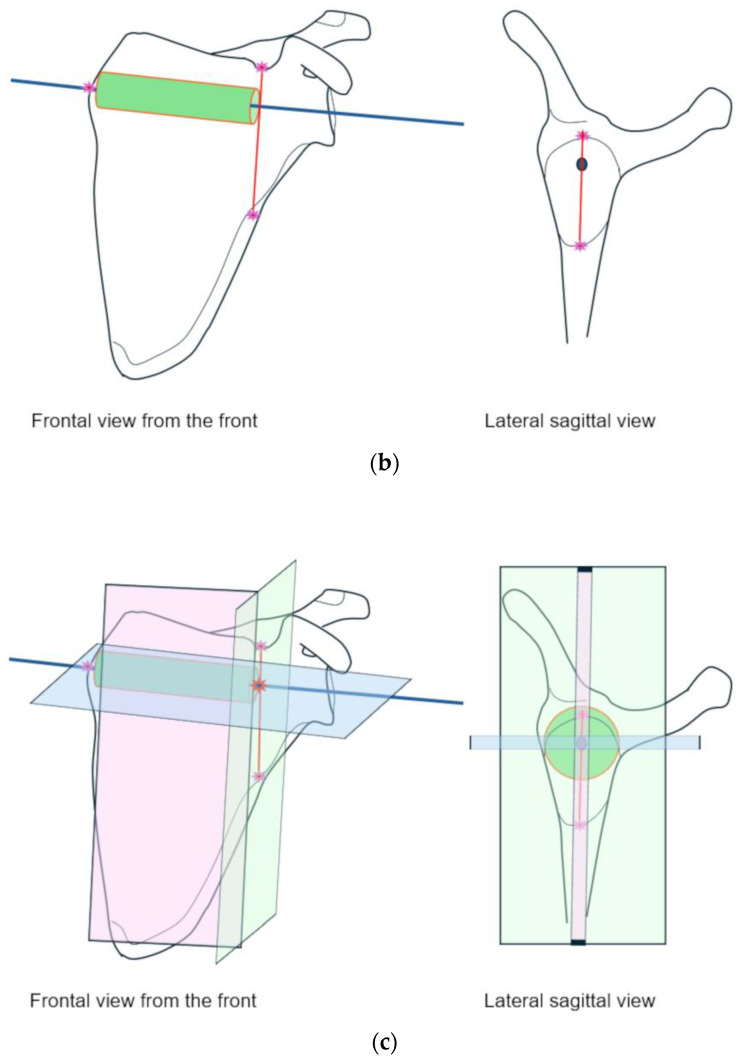
Schematic representation of the development of a three-dimensional measurement model of the scapula. Left scapula seen from the front in the image on the left and from the side in the image on the right. (**a**) A cylinder is depicted at the junction of the upper and lower bodies of the scapula and the scapular spine with a central axis. This axis is part of the axial plane. (**b**) Establishment of reference points on the frontal plane: one medial point corresponding to the most medial bone point on the axis. Two lateral points, at the apexes of the convexity of the middle of the scapular notch and the lateral lower edge, with the line connecting the two points passing through the axis of the cylinder and being parallel to the surface of the cylinder. (**c**) The pink plane is the frontal plane defined by the three points described above. The green plane lies in a plane parallel to the surface of the cylinder and constitutes the sagittal plane. The two planes are orthogonal. The blue plane is the axial plane, which is orthogonal to the previous two. The center of the coordinate system is the point where the three planes meet on the central axis (red star).

The following subsections address complementary and partly overlapping methodological factors, including landmark dependency, dimensional simplification, reference-plane alignment, and imaging variability, which collectively influence the reproducibility of acromial morphology measurements. This section provides a conceptual synthesis rather than an empirical validation. Figure 2, Figure 3, Figure 4 and Figure 5 serve as conceptual schematics illustrating methodological and geometric dependencies discussed in this section and are not intended as quantitative validation.

### 4.1. Inter-Study Reproducibility and Overlap of Values

The first limitation of the current literature is the wide variability in reported values across studies. Sagittal acromial tilt, for instance, ranges from 55.2° in posterior instability [6] to 78.8° in constitutional static instability [9]. Posterior coverage and acromial height show similar inconsistencies. These variations lead to substantial overlap between pathological and control groups, even when statistical significance is achieved. For example, Beeler et al. [8] demonstrated within their own series that instability and control shoulders displayed overlapping distributions of tilt and coverage, underlining the limited discriminatory power of these parameters. Such findings highlight that acromial measurements, although useful at the group level, cannot reliably distinguish pathology on an individual basis.

### 4.2. Influence of Anatomical Landmarks

A second major source of heterogeneity lies in the choice of reference landmarks. Many studies define tilt and coverage relative to the glenoid center or axis [8,11]; Verhaegen et al. [12]; Livesey et al. [7], yet this reference may be pathologically altered. In eccentric osteoarthritis, the glenoid is often eroded and retroverted; in posterior instability, it is frequently decentered. As schematically shown in Figure 2, translations of the glenoid center artificially affect sagittal tilt, posterior coverage and height, while Figure 3 illustrates how changes in glenoid version rotate the reference axis itself, modifying tilt values even if the acromion has not changed. These factors explain why studies using different reference definitions often produce contradictory results.

### 4.3. Two-Dimensional Simplifications of Three-Dimensional Structures

Several studies rely on 2D radiographs (Meyer et al., 2019 [5,10]) or sagittal MRI planes (Arner et al. [6]; Livesey et al. [7]), while even CT-based series (Beeler et al. [8]; Akgun et al. [9]) often collapse data into 2D projections for angular calculations. However, the acromion, glenoid, and scapula are inherently three-dimensional, non-coplanar structures. As demonstrated by Stehle et al. [13] and in Figure 4, small variations in scapular orientation or malrotation can cause significant shifts in measured tilt and coverage. The reliance on 2D simplifications therefore reduces accuracy and reproducibility across studies.

### 4.4. Imaging Modality and Technical Variability

The choice of imaging technique also impacts reported measurements. Radiographs are prone to calibration and projection errors. MRI measurements depend heavily on slice alignment, which varies between studies (aligned to the glenoid vs. the scapular axis). CT reconstructions differ according to whether they are multiplanar, manual, or based on statistical shape models. This explains why acromial height values reported by Arner et al. [6] are consistently lower than those of Meyer et al., 2019 [5] or Beeler et al. [8]. As highlighted in Table 2, methodological heterogeneity is at least as influential as true anatomical variation.

### 4.5. Statistical Limitations

Several series suffer from small sample sizes (Akgun et al. [9]; Meyer et al. [10]) or selective recruitment. Reporting formats are inconsistent: some authors present means with standard deviations, others use medians with interquartile ranges, and only a minority provide confidence intervals. These discrepancies further complicate inter-study comparisons and limit meta-analytical synthesis. Furthermore, interobserver reliability and measurement error were inconsistently reported across studies, further limiting reproducibility assessment and comparability of results.

### 4.6. Clinical Interpretability

From a clinical perspective, these methodological issues mean that posterior acromial parameters cannot currently be used as diagnostic cut-offs. A tilt of 63°, for example, may indicate instability in one study, eccentric osteoarthritis in another, or a normal shoulder in controls. At present, these parameters should be considered population-level risk markers rather than individual diagnostic tools.

### 4.7. Toward Standardized 3D Reference Models

Faced with these limitations, a methodological shift appears indispensable. Rather than relying on glenoid-based landmarks that may be altered by pathology, it is more appropriate to adopt stable anatomical reference points unaffected by degenerative change. Several authors have already proposed alternative reference axes, notably through statistical shape models or three-dimensional volumetric analyses in the evaluation of glenoid version [15,16,17,18]. Building on these approaches, acromial morphology could be redefined using extrinsic scapular landmarks, such as the junction between the scapular spine and the anterior and posterior columns of the scapular body.

In this context, we propose a stable three-dimensional coordinate system derived from a “reference cylinder” centered on the internal cortices of the scapula at the level of the scapular notch (Figure 5). This model would allow both the acromion and glenoid to be positioned within a constant orthonormal reference frame, regardless of articular deformation or osseous wear. The potential advantages are twofold: first, to improve reproducibility across future morphometric studies; and second, to enable vector-based analyses that more faithfully capture the true spatial relationships between scapular structures [15,16,19]. Integrating such methods with dynamic and functional imaging may ultimately clarify whether acromial orientation represents a predisposing factor or an adaptive remodeling phenomenon.

## 5. Discussion

The posterior acromion has gained increasing attention as a potential factor in the pathogenesis of chronic shoulder disorders. Beyond its classical implication in impingement and rotator cuff pathology, several studies have suggested that posterior acromial orientation may influence the development of posterior instability and eccentric glenohumeral osteoarthritis [5,6,7,8,9,10,11,12]. The synthesis of available data indicates that in these conditions the acromion tends to be more horizontally inclined, less posteriorly covering, and positioned higher relative to the scapular plane. In contrast, in massive RCTs, the acromion has been described as more posteriorly covering, without consistent changes in tilt [14]. These differing morphologic patterns suggest that the posterior acromion may interact with shoulder biomechanics in complex and potentially pathology-specific ways.

Biomechanically, a more horizontal and higher acromion may reduce posterior containment of the humeral head, facilitating posterior translation during motion and contributing to instability and eccentric wear. Conversely, increased posterior coverage may shift contact pressures anteriorly, potentially predisposing to cuff overload. These shape-related variations may therefore modify scapulohumeral kinematics and glenohumeral load distribution, supporting the concept that acromial morphology plays an active role in shoulder biomechanics.

Despite the recurrence of these trends, the variability across studies prevents their application as reliable diagnostic markers. As summarized in Table 1 and Table 2, absolute values of tilt, coverage, and height differ substantially depending on study design, imaging modality, and definitions. Standard deviations are wide, and reported ranges often overlap between pathological and control shoulders. For the clinician, this means that an individual measurement cannot be interpreted as pathognomonic of instability, osteoarthritis, or cuff failure. At present, posterior acromial parameters should be regarded as population-level associations rather than individual diagnostic thresholds.

These limitations reflect, in large part, methodological heterogeneity. Differences in imaging modality—ranging from plain radiographs to MRI and 3D reconstructions—produce non-comparable datasets. Definitions of anatomical landmarks also vary, with many studies using the glenoid center as a reference point despite its frequent alteration in pathological shoulders. In cases of posterior subluxation arthropathy or posterior instability, for instance, the geometric center of the glenoid (Walch type B) may be displaced due to retroversion, posterior glenoid wear, or humeral subluxation [10,12,20,21,22]. Using this landmark as a reference in measurement models therefore falsely assumes anatomical stability regardless of the clinical context. The reduction of three-dimensional scapular anatomy into two-dimensional projections further compounds these inconsistencies. Such methodological diversity undermines reproducibility and explains why results across series are not always concordant.

Nevertheless, the consistent directional trends across different clinical entities support the hypothesis that posterior acromial morphology contributes to shoulder pathology. Whether these changes represent a true etiological factor predisposing to instability and eccentric wear, or instead a secondary adaptation to chronic altered mechanics, remains uncertain. Clarifying this distinction is essential to determine whether acromial orientation could one day become a therapeutic target or should simply be considered a consequence of disease progression. However, the absence of longitudinal studies limits the ability to determine whether these morphological variations are predisposing factors or adaptive consequences of chronic pathology.

Future research should prioritize reproducibility and clinical applicability. Standardized, pathology-independent, three-dimensional reference systems, as proposed in methodological studies, are necessary to reduce bias and enable inter-study comparison. Integrating such morphometric approaches with dynamic imaging and kinematic assessment would help determine whether acromial orientation actively contributes to pathological loading of the glenohumeral joint or represents an adaptive remodeling phenomenon.

From a clinical standpoint, incorporating standardized acromial measurements into preoperative imaging protocols could enhance recognition of pathological scapular alignment and aid surgical decision-making. Three-dimensional reference models may facilitate objective assessment of acromial orientation during planning of reconstructive procedures, such as osteotomies or shoulder arthroplasty, and could support postoperative evaluation of joint centering and load redistribution.

Finally, the clinical relevance of posterior acromial morphology should not be underestimated. Beyond static morphometric descriptions, it would be valuable to complement these analyses with functional studies that integrate dynamic parameters such as joint kinematics or motion-based imaging, in order to better understand the relationship between form and function. Such an integrated approach could open the way to predictive tools, useful both for diagnosis and for therapeutic decision-making in chronic shoulder disorders. In this context, Gerber et al. recently reported the case of a patient with static posterior shoulder subluxation treated with a combined acromial and glenoid osteotomy (SCOPE procedure) designed to restore physiological scapular morphology. Correction of a high, horizontal acromion together with a retroverted glenoid achieved durable joint centering and full functional recovery at two-year follow-up. Although anecdotal, this observation supports the hypothesis that acromial morphology may actively influence posterior shoulder stability, beyond the purely passive role long assumed [23].

This review has certain limitations inherent to its narrative design. The literature selection was intentionally focused rather than exhaustive, emphasizing studies that applied comparable measurement methodologies to ensure consistency in interpretation. As this work was conceived as a critical review rather than a systematic review, no formal quantitative quality or bias assessment was performed, since the primary objective was to highlight methodological trends and reproducibility considerations rather than to statistically weight evidence.

## 6. Conclusions

Posterior acromial morphology appears to influence shoulder biomechanics, with instability and eccentric osteoarthritis associated with a more horizontal, less covering, and higher acromion, and massive cuff tears linked to increased posterior coverage. Despite these associations, methodological variability and overlapping values limit their diagnostic application. Future studies should establish standardized imaging acquisition and reconstruction protocols (CT/MRI) with uniform, pathology-independent reference systems anchored to stable scapular landmarks and validated across modalities. Prospective, multicenter validation studies incorporating predefined interobserver reliability thresholds and measurement-error reporting will be essential to enhance reproducibility and clinical applicability. Together, these efforts may ultimately establish posterior acromial orientation as a clinically meaningful parameter and potential therapeutic target.

## Figures and Tables

**Table 1:** Characteristics of included studies evaluating posterior acromial morphology.

Author (Year)	Pathology/Subgroups (*n*)	Imaging Modality	Parameters Reported	Key Findings	*p* *
Meyer et al. (2019) [5]	Posterior instability (41), Anterior instability (41), Healthy controls (53)	Radiograph (Y-view)	Tilt, coverage, height	Instability group: greater tilt and height, reduced coverage vs. controls	0.001
Beeler et al. (2021) [8]	Posterior instability—dynamic (30), static (20), Controls (40)	CT (3D reconstructions)	Tilt, coverage, height	Instability: greater tilt and height, reduced coverage vs. controls; overlap shown (Figure 1)	0.004
Akgun et al. (2024) [9]	Constitutional static posterior instability C1 (17 shoulders from 10 pts), Controls (68 shoulders from 40 pts)	MRI (3D reconstructions), PET-CT (controls)	Tilt, coverage, height	Instability: markedly increased tilt and height, reduced coverage vs. controls	0.001
Livesey et al. (2023) [7]	Instability without bone loss (30), with <13.5% bone loss (45), with >13.5% bone loss (14)	MRI (sagittal plane aligned to glenoid)	Tilt, coverage, height	Tilt and height increased with bone loss; reduced coverage only in pooled analysis	0.004
Arner et al. (2023) [6]	Posterior instability (46), Anterior instability (99), Controls with snapping scapula (64)	MRI	Tilt, coverage, height	Instability: lower tilt and height increased, reduced coverage vs. controls	0.026
Meyer et al. (2019) [10]	Concentric OA Walch A–B (77), Eccentric OA Walch B2–C (22)	Radiograph (Y-view), CT (multiplanar)	Tilt	Greater tilt in eccentric vs. concentric OA	0.004
Beeler et al. (2018) [11]	Concentric OA (45), Eccentric OA (65)	CT (multiplanar reconstructions)	Tilt, coverage	Eccentric OA: greater tilt, reduced coverage vs. concentric OA	0.002
Verhaegen et al. (2021) [12]	OA (117), Controls (110)	CT (statistical shape model)	Tilt, coverage	OA: greater tilt, reduced coverage vs. controls	<0.001
Beeler et al. (2018) [14]	Massive cuff tear (70), Concentric OA (45)	CT (multiplanar reconstructions)	Tilt, coverage	Cuff tears: greater posterior coverage, no tilt difference vs. OA	0.01 ^

Abbreviations: OA: osteoarthritis; MRI: magnetic resonance imaging; CT: computed tomography; 3D: three-dimensional; PET-CT: positron emission tomography; Pts: patients, *p* *: *p*-value for sagittal tilt; ^: *p*-value for coverage.

**Table 2:** Quantitative results of posterior acromial parameters across included studies.

Author (Year)	Pathology/Subgroup (*n*)	Sagittal Tilt (°) Mean ± SD	Posterior Coverage (°) Mean ± SD	Acromial Height (mm) Mean ± SD
Meyer et al. (2019) [5]	Posterior instability (41)	63.6 ± 9.8	48.8 ± 8.5	30.9 ± 6.7
	Anterior instability (41)	55.8 ± 8	60.9 ± 11.6	19.5 ± 8.5
	Controls (53)	55.9	61.6	20.4
Arner et al. (2023) [6]	Posterior instability (46)	55.2 ± 9.6	68.3 ± 11.0	11.0 ± 5.5
	Anterior instability (99)	56.8 ± 8.5	88.7 ± 10.8	−0.1 ± 5.1
	Controls (64)	62.2 ± 13.7	81.7 ± 19.1	0.7 ± 9.8
Livesey et al. (2023) [7]	Instability—no bone loss (30)	58.5 ± 1.4	65.4 ± 1.7	17.6 ± 1.3
	Instability—<13.5% bone loss (45)	64.3 ± 1.5	60.0 ± 1.4	20.4 ± 1.1
	Instability—>13.5% bone loss (14)	67.7 ± 1.8	61.1 ± 3.4	21.9 ± 2.5
Beeler et al. (2021) [8]	Static instability (20)	63 ± 8.5	54.6 ± 6.7	21.3 ± 4.2
	Dynamic instability (30)	59.3 ± 9.3	56.7 ± 9.5	20.3 ± 5.6
	Controls (40)	55.7 ± 7.6	62.9 ± 7.5	15.5 ± 4.9
Akgun et al. (2024) [9]	Constitutional static instability C1 (17)	78.8 ± 5.0	52.8 ± 11.1	21.9 ± 4.6
	Controls (68)	68.8 ± 3.9	63.2 ± 6.3	18.6 ± 4.7
Meyer et al. (2019)	Eccentric OA B2–C (22)	61.0 ± 9.0	-	-
[10]	Concentric OA A–B (77)	56.0 ± 11.0	-	-
Beeler et al. (2018) [11]	Eccentric OA (65)	72.0 ± 7.9	53.0 ± 6.9	-
	Concentric OA (45)	67.0 ± 7.3	57.0 ± 5.8	-
Verhaegen et al. (2021) [12]	OA (117)	70.0 ± 9.0	50.0 ± 8.0	-
	Controls (110)	64.0 ± 8.0	55.0 ± 7.0	-
Beeler et al. (2018) [14]	Cuff tear (70)	64.5 ± 7.0	60.0 ± 8.6	-
	Concentric OA (45)	67.2 ± 7.5	54.5 ± 5.9	-

Abbreviations: SD: standard deviation; OA: osteoarthritis.

**Table 3:** Summary of overall trends in posterior acromial morphology across pathologies.

Pathology	Tilt	Coverage	Height
Posterior instability	↑ (more horizontal) *	↓	↑
Eccentric GH osteoarthritis	↑	↓	-
Massive cuff tears	≈	↑	-

*↑ = increased; ↓ = reduced; ≈ = no significant change; – = not evaluated.* * Exception: Arner et al. (2023) [6] reported lower tilt values in posterior instability compared with controls. Abbreviations: GH: glenohumeral.
